# Development and application of “Special defibrillator for teaching and training”

**DOI:** 10.1186/s40001-022-00657-4

**Published:** 2022-03-02

**Authors:** Liping Xing, Shujing Wu, Xuemei Zhong, Zhisheng Duan, Fengzhen Wang, Zhiyou Liu, Liang Nie, Hongying Xie

**Affiliations:** 1 Department of Critical Care, The First Affiliated Hospital of Gannan Medical University, No. 123 Jinling Avenue, Zhanggong District, Jiangxi, 341000 China; 2 Department of Nursing, Gannan Health Vocational College, Jiangxi, 341000 China; 3 Department of Breast Disease Prevention and Control Center, Guangdong Women and Children Hospital, Guangdong, 511400 China; 4 Department of Cardiac Intervention Room, The First Affiliated Hospital of Gannan Medical University, Jiangxi, 341000 China

**Keywords:** Special defibrillator for teaching and training, Development, Application, Ventricular fibrillation

## Abstract

**Background:**

To provide an economical and practical defibrillator for first aid teaching and training, to reduce the cost of teaching and training, increase teaching and training equipment, provide trainees with more hands-on training sessions, and improve first aid capabilities.

**Methods:**

Developing a special teaching defibrillator with the same structure and operation configuration as the clinical medical emergency defibrillator. The appearance, structure and operating accessories of the two defibrillators are the same. The difference between the defibrillator and the clinical medical emergency defibrillator are as follows: the clinical medical emergency defibrillator can be energized, and there are expensive electronic accessories and defibrillation accessories for charging and discharging in the machine. When discharging, the electrode plate has current discharged into the human body; the power plug of the “special defibrillator for teaching and training” is a fake plug. When the power is plugged in, no current enters the body and the machine. There are no expensive electronic accessories and defibrillation accessories for charging and discharging, and no current is discharged during discharge. Then compare the teaching effect of the special defibrillator for teaching and training and the clinical medical emergency defibrillator (including operation score and attitude after training).

**Results:**

The scores of defibrillator operation in the experimental group of junior college students (87.77 ± 4.11 vs. 83.30 ± 4.56, *P* < 0.001) and the experimental group of undergraduate students (90.40 ± 3.67 vs. 89.12 ± 3.68, *P* = 0.011) were higher than those in the corresponding control group; The attitude of junior college students in the experimental group and undergraduate students in the experimental group after training was more positive than that of the corresponding control group (*P* < 0.05).

**Conclusions:**

The special defibrillator for teaching and training can save the purchase cost of teaching equipment, increase teaching and training resources, and improve the trainee’s defibrillation ability, defibrillation confidence and defibrillation security.

## Background

The number of people who suffer from cardiac arrest each year is as high as 8–9 million people in the world [1], 75% of which are sudden cardiac death (SCD). The number of people who suffer from SCD each year in China is about 544,000, ranking first in the world [2]. 90% of SCD results from fatal arrhythmias, of which ventricular fibrillation accounts for 80%. Electrical defibrillation is the most effective way to terminate ventricular fibrillation [3]. Studies have reported that the probability of survival to discharge from a cardiac arrest is about 15–25%, and it has increased in the past 10–15 years [4–6]. It has been shown that the delay in defibrillating patients with severe congenital heart disease has a negative impact on the probability of survival to discharge [7]. Therefore, timely response to cardiac arrest is essential to improve the patient’s prognosis. The American Heart Association resuscitation and cardiovascular emergency guideline 2020 emphasizes that patients with cardiac arrest whose initial rhythm is defibrillator rhythm should be given electric defibrillation as soon as possible [8]. For every 1-min delay of defibrillation, the patient’s long-term survival rate will be reduced by 1% [9], early electrical defibrillation is the key to successful patient resuscitation.

In recent years, people pay more attention to the school education of automatic external defibrillator (AED) [10, 11]. For patients with in-hospital ventricular fibrillation, medical staff are the first witness, and their defibrillation technology and ability are important factors to determine the success rate of resuscitation and survival rate of patients with ventricular fibrillation. Therefore, strengthening the defibrillation skills training of medical staff is the focus of hospital quality management. However, due to the high price of current clinical medical emergency defibrillators, there is a shortage of defibrillators in various medical schools and hospitals. It is difficult for school students and medical staff to have practical training opportunities when receiving emergency defibrillation training [12, 13]. Therefore, this research aims to develop a kind of “special defibrillator for teaching and training” (Patent No. zl2014-20411878.9), which is cheap, durable, and can meet the needs of defibrillation skills operation process training and easy to manage.

## Methods

### Structure comparison and usage method of two defibrillators

**Fig. 1:**
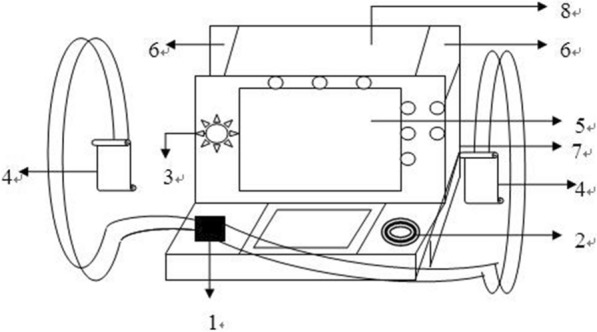
The structure and operating accessories of the two defibrillators

The structure and operating accessories of the two defibrillators are the same, as shown in Fig. 1.

The two defibrillators have a shell, on which the lead wire insertion port of defibrillation electrode plate, the lead wire insertion port of simulated electrocardiogram and the charging energy selection switch are set; a simulated electrocardiogram display screen is also installed on the shell, and a defibrillation electrode plate placing area is arranged at the upper rear of the left and right sides of the ECG display screen on the shell; two defibrillation electrode plates and a discharge switch are connected to the front end of the defibrillation electrode plate lead wire.

### The difference between the two defibrillators

The clinical medical emergency defibrillator can be energized, and there are expensive electronic accessories and defibrillation accessories for charging and discharging in the machine. When discharging, the electrode plate has current discharged into the human body; the power plug of the “special defibrillator for teaching and training” is a fake plug. When the power is plugged in, no current enters the body and the machine. There are no expensive electronic accessories and defibrillation accessories for charging and discharging, and no current is discharged during discharge.

### Usage of two defibrillators

The operation process of the two defibrillators is the same. Plug in the power plug → turn on the power switch → stick the ECG electrode piece on the right shoulder, left shoulder and left upper abdomen according to the lead label → check whether there are metal objects on the patient’s body → apply conductive glue on the electrode plate → select energy → place the sternum electrode plate on the second intercostal of the right edge of sternum and the apex electrode plate on the fifth intercostal of the left axillary midline → ask others to leave the bed edge → press the electrode plate with both arms straight and press tightly to the patient’s skin → keep the operator away from the edge of the bed → press the charging switch → press the discharge switch → check the patient’s skin → tidy up the patient’s clothes and bed sheets → tidy up other objects.

### Application effect evaluation

#### Object

A total of 218 students in the nursing undergraduate class (undergraduate group) of a medical college in Jiangxi Province and 218 students in the nursing junior college class (junior college group) of a health vocational college in Jiangxi Province were selected as the research objects. In the junior college group, there were 208 female students and 10 male students; they are 18–25 years old. There are 208 female students and 10 male students in the undergraduate group; they are 18–25 years old. In the junior college group, 110 people were randomly selected as the junior college observation group, and the remaining 108 persons were the junior college control group. In the undergraduate group, 110 people were randomly selected as the undergraduate observation group, and the remaining 108 people were in the undergraduate control group. This study was approved by the ethics committee of The First Affiliated Hospital of Gannan Medical University.

#### Teaching methods

Set up a training assessment team, with members of the research group as team members. The theoretical training contents and operation procedures of defibrillation were formulated according to the syllabus of “Emergency and Critical Care Nursing” [14]. Train and evaluate the members of the research group before the student training, and only after passing the examination can they post teaching, to ensure the homogenization of the training of teachers. The total training time was 1 class hour (40 min), and practice in groups 2 class hours after training.

### Teaching methods of the control group

#### Teaching mode

The mode of combining theory with practice teaching was adopted.

#### Theoretical teaching

The teacher prepared lessons and made PPT on the theoretical content of defibrillation according to the requirements of the syllabus. In the course of classroom teaching, the learning purpose, process, and key points of the defibrillator were explained for about 25 min.

#### Operation training

Clinical medical first aid defibrillator was used for training by teachers. The time was 15 min. Finally, students practiced the use of clinical medical emergency defibrillators in groups, and could repeatedly invite teachers to answer questions, guide, demonstrate, and correct operations according to their own needs. The time was about 80 min.

### Teaching methods of the experimental group

#### Teaching mode

The mode of combining theory with practice teaching was adopted.

#### Theoretical teaching

The teacher prepared lessons and made PPT on the theoretical content of defibrillation according to the requirements of the syllabus. In the course of classroom teaching, the learning purpose, process, and key points of the defibrillator were explained for about 25 min.

#### Operation training

A special defibrillator for teaching was used for training by teachers. The time was 15 min. Finally, students practiced the use of clinical medical emergency defibrillators in groups, and could repeatedly invite teachers to answer questions, guide, demonstrate, and correct operations according to their own needs. The time was about 80 min.

### Effect evaluation index

#### Operation score

All the students were assessed with clinical application defibrillators. Two teachers scored according to the latest defibrillator operation scoring standard recommended by the American Heart Association 2015 edition of “International Cardiopulmonary Resuscitation and Cardiovascular First Aid Guide” [15], and the average value was calculated as the final result. The operation scores were immediately collected after the training.

#### Training attitude

After the training was completed, each student needed to complete a “Defibrillator Training Attitude Questionnaire” compiled by the research team. The questions included: “Through training, my external defibrillation skills have improved.”, “When a patient has a sudden ventricular fibrillation, I am confident to implement external defibrillation for him/her.”, “During the training, I worry that the current will hurt myself or others due to wrong operation.”, and “This training method has improved my hands-on ability.”. Each question had two options: “Agree” and “Disagree”. The number of people who chose “agree” in each question of each group was counted.

#### Statistical methods

IBM SPSS25.0 statistical software was used for analysis. The measurement data conforming to the normal distribution were statistically described by the mean ± standard deviation, and the *t*-test was used for the comparison between the groups; the counting data were statistically described by frequency or constituent ratio. Chi square test or Fisher’s exact test was used for comparison between the groups. *P* < 0.05 indicated that the difference was statistically significant.

## Results

There was no statistically significant difference between the observation group and the control group of junior college and undergraduate students in terms of number, age, final grades of last semester, and results of defibrillator operation before training (*P* > 0.05). See Tables 1 and 2 for details.

**Table 1:** Comparison of general information of two groups of undergraduate students

Group	Number of people	Age	Final exam results	Results of defibrillator operation before training
Undergraduate observation group	110	21.8 ± 1.7	83.7 ± 5.6	85.4 ± 4.9
Undergraduate control group	108	21.4 ± 1.7	83.7 ± 5.6	85.2 ± 4.9
*t*		− 8.57	− 0.17	0.213
*P*		0.223	0.986	0.832

**Table 2:** Comparison of general information of two groups of junior college students

Group	Number of people	Age	Final exam results	Result of defibrillator operation before training
Junior college observation group	110	21.7 ± 1.7	83.4 ± 5.6	85.4 ± 4.4
Junior college control group	108	21.3 ± 1.4	83.3 ± 5.6	85.6 ± 4.2
*t*		19.467	0.064	− 3.78
*P*		0.109	0.949	0.706

The defibrillator operation scores of the experimental group of junior college students (*P* < 0.001) and the experimental group of undergraduate students (*P* = 0.011) were higher than those of the corresponding control group, as shown in Tables 3 and 4.

**Table 3:** Comparison of the scores of the external defibrillation operation test between the two groups of junior college students after training

Group	Number of people	Score
Experimental group	110	87.77 ± 4.11
Control group	108	83.30 ± 4.56
*t*		7.607
*P* value		0.000

**Table 4:** Comparison of the scores of the external defibrillation operation test between the two groups of undergraduate students after training

Group	Number of people	Score
Experimental group	110	90.40 ± 3.67
Control group	108	89.12 ± 3.68
*t*		2.57
*P* value		0.011

Compared with the control group, the experimental group of junior college students and the experimental group of undergraduate students had a more positive attitude after training, and their defibrillation self-confidence was improved (*P* < 0.05); see Tables 5 and 6.

**Table 5:** Post-training attitude of junior college students

Item	I	II	III	IV
Junior college experimental group	90	72	18	89
Junior college control group	60	50	40	52
Statistical value (*I*^2^)	17.513	8.116	11.927	25.602
*P*	0.000	0.004	0.001	0.000

I, through training, my external defibrillation skills have improved; II, when a patient has a sudden ventricular fibrillation, I am confident to implement external defibrillation for him/her; III, during the training, I worry that the current will hurt myself or others due to wrong operation; IV, this training method has improved my hands-on ability

**Table 6:** Post-training attitude of undergraduate students

Item	I	II	III	IV
Undergraduate experimental group	102	87	5	109
Undergraduate control group	78	64	13	100
Statistical value (*I*^2^)	15.920	10.068	4.038	–
*P*	0.000	0.002	0.044	0.018

The data do not meet the conditions of Chi square test, and Fisher exact test method is used for comparison. I, through training, my external defibrillation skills have improved; II, when a patient has a sudden ventricular fibrillation, I am confident to implement external defibrillation for him/her; III, during the training, I worry that the current will hurt myself or others due to wrong operation; IV, this training method has improved my hands-on ability

## Discussion

The results of this study show that after training junior college and undergraduate medical students with the “special defibrillator for teaching and training” and manual defibrillator, the defibrillation skills and defibrillation self-confidence of the four groups of students have been improved, which is consistent with the results of similar studies [16]. After the theoretical explanation, students integrate theoretical cognition and action skill training into the operation process in the simulation practice process, master and use the knowledge learned to obtain skills and experience, thus improving their operation skills and self-confidence [17]. The results show that there is no significant difference between the experimental group and the control group in the operational performance and operational confidence of the students, suggesting that the “special defibrillator for teaching and training” and the clinically applied manual defibrillator have the same training effect in operational training.

Students were highly satisfied with the training based on the “special defibrillator for teaching and training”. The combination of theory and practice is the most common teaching method in clinical practice. In this study, students showed a high degree of satisfaction with the mixed teaching based on the combination of the theory of “special defibrillator for teaching and training” + practical exercises. Weitong et al. [18] believe that the degree of interaction and learning achievement have a direct positive impact on training satisfaction. Through the combined training of theory + practical exercises [19], students can immediately train in groups under the guidance of the teacher after listening to the theoretical knowledge. They can master practical skills to the greatest extent, improve their knowledge and skills, and have a sense of learning achievement. Moreover, they can communicate with teachers and classmates, which improves their interest in learning. In addition, this study also found that students who used the “special defibrillator for teaching and training” for training expressed lower safety concerns. The reason may be that the mechanical structure of the “special defibrillator for teaching and training” does not have electronic accessories and charging and discharging modules, but it has almost the same appearance and use feeling as the clinical manual defibrillator, and also avoids the hidden dangers of safety risks and damage risks. For beginners, the “special defibrillator for teaching and training” can eliminate students’ worries and concerns in the process of practice, ensure the safety of practice, and enable them to participate in the operation practice more actively. “Special defibrillator for teaching and training”, as a kind of simulation teaching equipment, can replace clinical application defibrillator for clinical and school teaching and training, and help to solve the current situation of insufficient training equipment.

At present, clinical application defibrillators are used in defibrillation teaching and training, but the number of defibrillators equipped in most hospitals, especially in primary hospitals and medical colleges and universities, is quite limited. In this study, 40 public hospitals above secondary level and 3 medical colleges and universities in the city were investigated, and it was found that only 7 hospitals were equipped with 1 defibrillator for training. Three of them used the discarded defibrillator for training; there were only nine defibrillators in three medical colleges, and only one defibrillator was reserved in two of them. Under this condition, it will inevitably lead to less training opportunities for students, which will greatly affect the quality of defibrillation training. Relevant investigations have also revealed this phenomenon. The medical staff in general clinical departments, especially the basic medical staff, have very little knowledge and skills in cardiac electrical defibrillation [8]. It can be seen that the shortage of defibrillator training equipment in hospitals, medical schools and so on is a problem that needs to be solved urgently [20]. The reasons may be as follows: (1) the price of the defibrillator supplied by the manufacturer is too expensive, and the hospital and the school have limited funds; (2) the defibrillator is equipped with electronic accessories, which need to be charged and quality checked regularly; the defibrillation electrode plate and the charge and discharge management module are scientifically and rigorously designed. Repeated or nonstandard operation can easily lead to defibrillator damage and high maintenance cost [21, 22].

“Special defibrillator for teaching and training” helps to solve the problem of insufficient defibrillator equipment through the following major advantages: (1) “Special defibrillator for teaching and training” is only used for teaching and training. It imitates clinical application defibrillator in appearance, only preserves its operation function, does not pursue the defibrillation performance of defibrillator, and has lower requirements in raw materials and manufacturing process, greatly reducing the production cost. (2) The clinical application defibrillator has high cost and easily damaged electronic accessories and charge–discharge modules, and it is easy to be damaged if the operation is not standardized. “Special defibrillator for teaching and training” is not designed with electronic accessories and charging and discharging modules. It is easy to maintain and repair, which is convenient for students to use repeatedly and boldly, and can save storage and maintenance costs. (3) “Special defibrillator for teaching and training” is not designed for power-on performance, and it is safer when used by students. After increasing the number of reserves, it can be used for students to operate and practice in a large range, shortening the training period and saving time and cost.

The disadvantage of this study is that we mainly compared the self-developed “special defibrillator for teaching and training” (Patent No. zl2014-20411878.9) with the clinical application defibrillator. It has not been compared with the existing defibrillators for teaching and training on the market to understand the advantages (such as teaching results and price) of the defibrillator for teaching and training in this study compared to other defibrillators for teaching and training.

## Conclusions

Defibrillator training based on “special defibrillator for teaching and training” improves students’ defibrillation skills and abilities, and has the same training effect as training based on clinically applied manual defibrillators. Moreover, it can save the cost of purchasing defibrillator and the human resources of equipment maintenance in hospitals and medical colleges, provide sufficient equipment resources for medical staff and school medical students to conduct defibrillation skills training, which is suitable for promotion and application.

## Data Availability

All data generated or analyzed during this study are included in this published article.

## References

[1] Li XY (2014). Summary of the experience of 138 cases of sudden death patients before hospitalization. China Pr Med.

[2] Fan YQ (2015). Design and key technology research of external automatic defibrillator.

[3] Liu GM (2019). Evaluation and decision-making of ventricular arrhythmia. Dig Latest World Med Inf.

[4] Morrison LJ, Neumar RW, Zimmerman JL (2013). Strategies for improving survival after in-hospital cardiac arrest in the United States: 2013 consensus recommendations: a consensus statement from the American Heart Association. Circulation.

[5] Peberdy MA, Kaye W, Ornato JP (2003). Cardiopulmonary resuscitation of adults in the hospital: a report of 14720 cardiac arrests from the national registry of cardiopulmonary resuscitation. Resuscitation.

[6] Girotra S, Nallamothu BK, Spertus JA (2012). Trends in survival after in-hospital cardiac arrest. N Engl J Med.

[7] Bircher NG, Chan PS, Xu Y (2019). Delays in cardiopulmonary resuscitation, defibrillation, and epinephrine administration all decrease survival in in-hospital cardiac arrest. Anesthesiology.

[8] He YR, Zheng Y, Zhou FT (2020). American heart association cardiopulmonary resuscitation and omitted guide interpretation adult basics advanced life support. West China Med J.

[9] Bircher NG, Chan PS, Xu Y (2019). American heart association’s get with the guidelines resuscitation investigators delays in cardiopulmonary resuscitation, defibrillation, and epinephrine administration all decrease survival in in-hospital cardiac arrest. Anesthesiology.

[10] Berger S, Utech L, Hazinski MF (2004). Lay rescuer automated external defibrillator programs for children and adolescents. Pediatr Clin N Am.

[11] Cave DM, Aufderheide TP, Beeson J (2011). Importance and implementation of training in cardiopulmonary resuscitation and automated external defibrillation in schools: a science advisory from the American Heart Association. Circulation.

[12] Hart D, Flores-Medrano O, Brooks S (2013). Cardiopulmonary resuscitation and automatic external defibrillator training in schools: “is anyone learning how to save a life?”. CJEM.

[13] Zinckernagel L, Hansen CM, Rod MH (2017). A qualitative study to identify barriers to deployment and student training in the use of automated external defibrillators in schools. BMC Emerg Med.

[14] Zhang B, Gui L. Emergency and Critical Care Nursing. People’s Medical Publishing House (PMPH); 2017.

[15] Kronick SL, Kurz MC, Lin S (2015). Part 4: systems of care and continuous quality improvement: 2015 American heart association guidelines update for cardiopulmonary resuscitation and emergency cardiovascular care. Circulation.

[16] Liu JJ, Gui L, Chen ZM (2015). The current status of defibrillation skills mastery of primary military doctors and the effect of automatic external defibrillator training. Nurs J Chin People's Lib Army.

[17] Xu HY, Wang BJ, Xu XJ (2020). Application of scenario simulation teaching in cardiovascular medicine resident omitted training cardiopulmonary resuscitation and electrical defibrillation teaching. Lab Med Clin.

[18] Liu WT, Wang XX (2019). Research on the influencing factors of blended teaching satisfaction. Mod Educ Technol.

[19] Vetter VL, Haley DM, Dugan NP (2016). Innovative cardiopulmonary resuscitation and automated external defibrillator programs in schools: results from the student program for Olympic resuscitation training in schools (SPORTS) study. Resuscitation.

[20] Liu CH, Sung CW, Fan CY (2021). Strategies on locations of public access defibrillator: a systematic review. Am J Emerg Med.

[21] Couloures KG, Allen C (2017). Use of simulation to improve cardiopulmonary resuscitation performance and code team communication for pediatric residents. MedEdPORTAL.

[22] Myers B, Obr C (2017). Preparing for cardiopulmonary bypass: a simulation scenario for anesthesia providers. MedEdPORTAL.

